# 2252. Characteristics and Management of non-ICU Patients with Community-Acquired Pneumonia (CAP)

**DOI:** 10.1093/ofid/ofad500.1874

**Published:** 2023-11-27

**Authors:** Matthew B Goetz, Valerie Vaughn, Christopher J Graber, Allison Kelly, Makoto M Jones

**Affiliations:** VA Greater Los Angeles Healthcare System, Los Angeles, California; University of Utah Medical School, Salt Lake City, Utah; VA Greater Los Angeles Healthcare System/UCLA, Los Angeles, California; US Department of Veteran Affairs, Cincinnati, Ohio; Veterans Affairs, Salt Lake City, Utah

## Abstract

**Background:**

Pneumonia is the most common reason for hospitalization and is often associated with excessively broad empiric therapy & long antibiotic duration. To evaluate overuse, we assessed the indications & frequency of MDRO therapy, risk factors & post-admission culture positivity for MDRO, time to stability and duration of therapy in hospitalized persons with CAP not receiving intensive care.

**Methods:**

We identified patients in the VA Corporate Warehouse with discharge ICD-10 codes consistent with pneumonia from 1/2022 to 3/2023. Exclusion criteria included < 2 days of antibiotic therapy, ICU admission, concurrent infections, > 14 days of antibiotic therapy & severe immunocompromise. Per ATS/IDSA guidelines, we allowed anti-MRSA therapy for patients with a prior positive MRSA culture, anti-pseudomonal (PSA) therapy for patients a prior positive PSA culture, and both anti-MRSA & anti-PSA therapy in patients with severe pneumonia (defined by > 1 ATS/IDSA minor criteria [mental status & x-ray data were not evaluable]) and intravenous antibiotics in the prior 3 months. Similarly, we assessed antibiotic duration and considered > 6 days inappropriate if patients were clinically stable (no more than one sign of instability) or discharged by day 5.

**Results:**

The median age of the 19223 patients was 75 (IQR 68,81); 4.6% were women. 42%, 35% & 22% of patients had CURB-65 scores of 0-1, 2 & 3-4. While 32% (6185/19223) of patients received empiric MDRO therapy, per ATS/IDSA guidelines, only 12% (2330 patients) warranted MDRO therapy (593 had severe disease with prior IV antibiotics, 1737 had prior positive MRSA [1381] and/or PSA [457] cultures). Overall, 90% (5600/6185) of patients who received MDRO therapy were potentially overtreated while 6.5% (1213/19233) were potentially undertreated (Figure 1). MDRO were recovered in 6.3% of cases (Table 1). Across all patients, 98% (18870) were stable by day 5, 42% (7997) of whom received >6 days of therapy (Figure 2).

Figure 1

**Figure:**
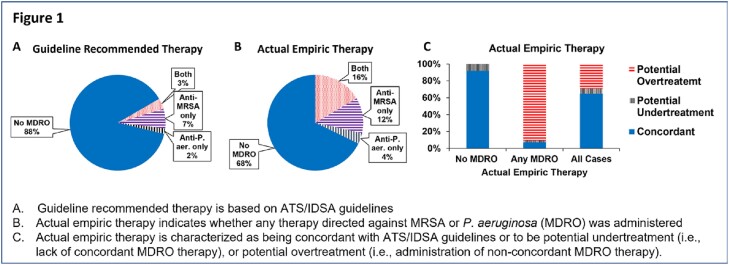

Table 1

**Figure:**
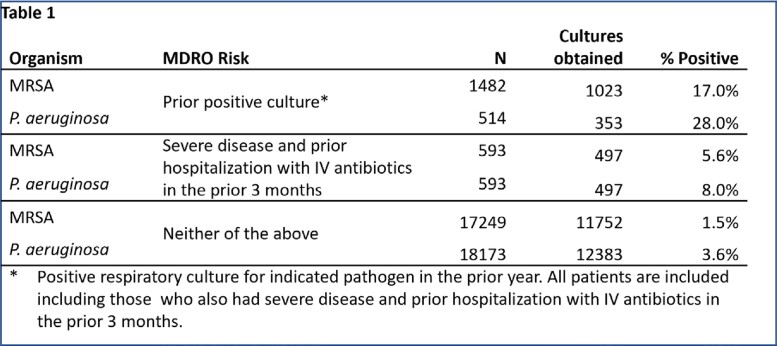

Figure 2

**Figure:**
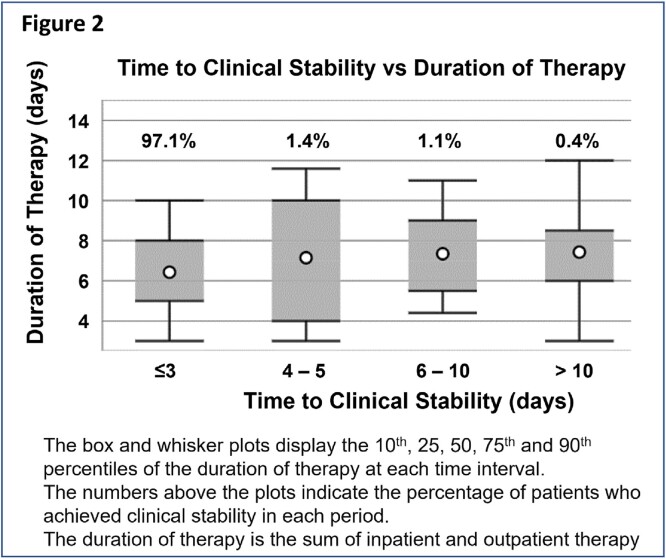

**Conclusion:**

We found substantial opportunities to reduce overuse of MDRO antibiotics and the duration of therapy in persons with CAP. Strengths of this analysis include the breadth and depth of the VA EHR. Limitations include the inability to assess selected measures of disease severity and stability (mental status changes and radiographic abnormalities).

**Disclosures:**

**All Authors**: No reported disclosures

